# Characterization of Integrin αIIbβ3-Mediated Outside-in Signaling by Protein Kinase Cδ in Platelets

**DOI:** 10.3390/ijms21186563

**Published:** 2020-09-08

**Authors:** Preeti Kumari Chaudhary, Sanggu Kim, Youngheun Jee, Seung-Hun Lee, Soochong Kim

**Affiliations:** 1 College of Veterinary Medicine, Chungbuk National University, Cheongju 28644, Korea; chaudharypreety11@gmail.com (P.K.C.); tkdrnfld@naver.com (S.K.); dvmshlee@cbu.ac.kr (S.-H.L.); 2 College of Veterinary Medicine and Veterinary Medical Research Institute, Jeju National University, Jeju 63243, Korea; yhjee@jejunu.ac.kr

**Keywords:** PKCδ, Pyk2, αIIbβ3, spreading, platelets

## Abstract

Engagement of integrin αIIbβ3 promotes platelet–platelet interaction and stimulates outside-in signaling that amplifies activation. Protein kinase Cδ (PKCδ) is known to play an important role in platelet activation, but its role in outside-in signaling has not been established. In the present study, we determined the role of PKCδ and its signaling pathways in integrin αIIbβ3-mediated outside-in signaling in platelets using PKCδ-deficient platelets. Platelet spreading to immobilized fibrinogen resulted in PKCδ phosphorylation, suggesting that αIIbβ3 activation caused PKCδ activation. αIIbβ3-mediated phosphorylation of Akt was significantly inhibited in PKCδ -/- platelets, indicating a role of PKCδ in outside-in signaling. αIIbβ3-mediated PKCδ phosphorylation was inhibited by proline-rich tyrosine kinase 2 (Pyk2) selective inhibitor, suggesting that Pyk2 contributes to the regulation of PKCδ phosphorylation in outside-in signaling. Additionally, Src-family kinase inhibitor PP2 inhibited integrin-mediated Pyk2 and PKCδ phosphorylation. Lastly, platelet spreading was inhibited in PKCδ -/- platelets compared to the wild-type (WT) platelets, and clot retraction from PKCδ -/- platelets was markedly delayed, indicating that PKCδ is involved in the regulation of αIIbβ3-dependent interactivities with cytoskeleton elements. Together, these results provide evidence that PKCδ plays an important role in outside-in signaling, which is regulated by Pyk2 in platelets.

## 1. Introduction

Integrins play a key role in regulating platelet functions, including platelet adhesion, spreading, clot retraction, and platelet pro-coagulant activity. Engagement of αIIbβ3 promotes platelet–platelet interaction [1] and generates outside-in signals that reinforce platelet activation [2]. Outside-in signaling in platelets is triggered when ligands such as fibrinogen bind to αIIbβ3, and it is dependent on close relationships between αIIbβ3 and the signaling machinery of the cell [3]. Upon activation of αIIbβ3, Src associates with β3 tails and integrin engagement increases Syk tyrosine phosphorylation in Src kinase-dependent manner [4,5,6]. Src and/or Syk phosphorylate several substrates, including SLP76, c-Cbl (adaptor protein), and Vav (a Rac GTPase), that have been implicated in signaling to the actin cytoskeleton [5,7,8]. The activation of Src kinase by αIIbβ3 also results in tyrosine phosphorylation of phospholipase Cγ2 (PLCγ2), which is required for lamellipodia formation [9,10]. Phosphoinositide 3-kinase (PI3K)/Akt also plays a role in signaling downstream of αIIbβ3 [1,11], and outside-in signaling also causes phosphorylation of focal adhesion kinase (FAK).

PKC is a serine/threonine-related protein kinase and has multiple isoforms classified to three subfamilies [12]. The conventional PKC (cPKC) isoforms include α, βI, βII, and γ and are activated by calcium and diacylglycerol (DAG), whereas novel PKC (nPKC) isoforms (-δ, -θ, -η, and -ε) are activated by DAG but not by calcium. Atypical PKC isoforms (ι, ζ) are not activated by calcium or DAG and require phospholipids for their activation. Functional isoforms α, β, δ, θ, η, ε, and ζ showed to be expressed in platelets [13,14] among PKC α, β, δ and θ, which are abundantly expressed in human platelets [15]. PKCδ is phosphorylated on Thr505 and has been implicated in negatively regulating glycoprotein (GP) VI-mediated platelet functional responses, whereas it positively regulated protease-activated receptor (PAR)-mediated platelet responses including granule secretion and thromboxane generation [16]. A previous study showed that PKCδ is a negative regulator of collagen-induced filopodia formation and platelet aggregation [17]. In addition, PKCδ was shown to be phosphorylated on tyrosine residues downstream of PAR and GPVI receptors [14,18]. PKCδ interacts specifically with Fyn tyrosine kinase downstream of the GPVI receptor, resulting in translocation of both kinases to the platelet membrane [18]. Phosphorylation of PKCδ on Y565 has been shown to potentiate the activity of the kinase [19], and PKCδ phosphorylated on Y311 has been implicated in mediating PAR-mediated thromboxane generation in platelets [14]. PKCδ also plays a role in megakaryopoiesis and is involved in the regulation of platelet leukocyte interaction during sepsis [20,21].

A previous study implicated PKC isoforms in the regulation of integrin properties in many cell types [22], but the role of specific PKC isoforms in platelet outside-in signaling is limited. It has also been reported that PKCβ-deficient platelets spread poorly on fibrinogen [15]. PKCθ has been reported to be tyrosine-phosphorylated during outside-in and GPVI signaling in platelets, and contributes to αIIbβ3-mediated outside-in signaling and actin-reorganization in platelets [23]. However, the role of PKCδ in the regulation of integrin-mediated outside-in signaling is not known. Earlier studies showed that PKCδ is activated by an integrin αIIbβ3-independent pathway [24]. PKCδ was reported to be involved in thromboxane A_2_ (TxA_2_) generation, and thrombin- and collagen-induced PKCδ phosphorylation is regulated by αIIbβ3 outside-in signaling, raising the potential role of PKCδ in αIIbβ3-mediated signaling [25]. Thus, our study was undertaken to determine the role of PKCδ and its signaling pathways in integrin-mediated outside-in signaling in platelets using PKCδ knockout mice.

In this study, we found that PKCδ is phosphorylated by integrin αIIbβ3-mediated outside-in signaling in platelets. We further found that integrin αIIbβ3-mediated PKCδ phosphorylation is regulated by Src and Pyk2, and αIIbβ3-mediated phosphorylation of Akt is regulated by PKCδ. Furthermore, PKCδ contributes to regulation of platelet spreading and clot retraction. Therefore, we concluded that PKCδ plays an important role in integrin αIIbβ3-mediated outside-in signaling in platelets.

## 2. Results

### 2.1. The Role of Integrin αIIbβ3-Mediated Signaling in PKCδ Phosphorylation

**Figure 1:**
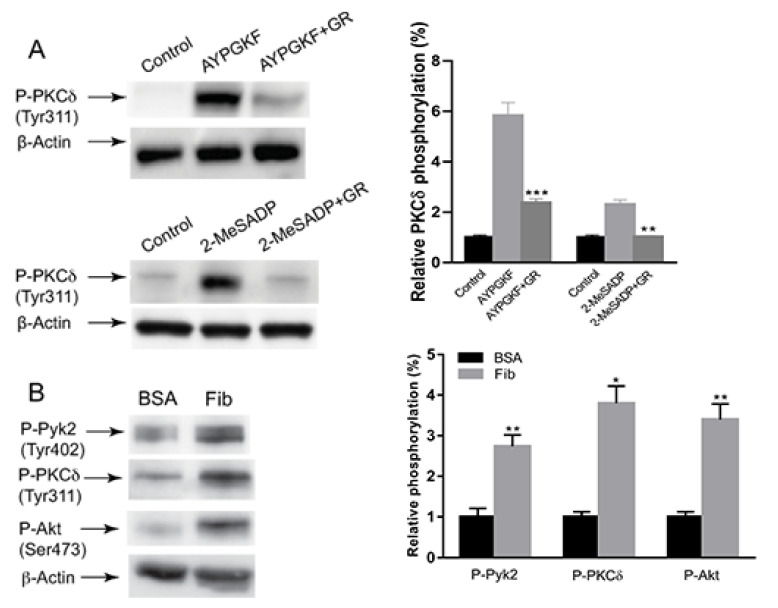
The effect of integrin αIIbβ3 on PKCδ phosphorylation in platelets. (**A**) Washed murine platelets were stimulated at 37 °C with 200 µM AYPGKF and 100 nM 2-MeSADP in the presence and absence of 1 µM GR144053 and probed with antibodies indicated. The blots are representative of three independent experiments and are presented as mean ± standard error (SE). ** *p* < 0.01 and *** *p* < 0.005. (**B**) Platelets were plated on either bovine serum albumin (BSA) (5 mg/mL) or fibrinogen (100 μg/mL) for 45 min, and lysates were probed with anti-phospho-Pyk2 (Tyr402), anti-phospho-PKCδ (Tyr311), anti-phospho-Akt (Ser473) or anti-β-actin (lane loading control) antibodies by western blotting. The blots shown are representative of three independent experiments and are presented as mean ± SE. * *p* < 0.05 and ** *p* < 0.01.

To investigate the signaling mechanism of PKCδ activation in outside-in signaling, we first evaluated the role of integrin αIIbβ3 in PKCδ activation. As shown in Figure 1A, AYPGKF-induced PKCδ (Tyr311) phosphorylation was significantly but not completely inhibited by a fibrinogen receptor antagonist GR144053. Similarly, 2-Methylthio adenosine diphosphate (2-MeSADP)-induced PKCδ (Tyr311) phosphorylation was completely inhibited in the presence of GR144053, suggesting that AYPGKF- and 2-MeSADP-induced PKCδ phosphorylation occurs mainly through integrin αIIbβ3-dependent pathways. To confirm the contribution of integrin αIIbβ3 to PKCδ phosphorylation, we investigated whether selective activation of integrin αIIβ3 can lead to the activation of PKCδ. There was an increase in phosphorylation of PKCδ upon platelet adhesion to immobilized fibrinogen, confirming that integrin αIIbβ3-mediated outside-in signaling leads to PKCδ phosphorylation. Platelet adhesion to fibrinogen also resulted in an increase in the phosphorylation of Pyk2 (Tyr402) and Akt (Ser473), which showed to be the downstream signaling events of integrin αIIbβ3 [26]. Thus, our data indicated that PKCδ is activated by integrin-mediated outside-in signaling in platelets.

### 2.2. αIIbβ3-Mediated PKCδ Phosphorylation is Regulated by Src and Pyk2

**Figure 2:**
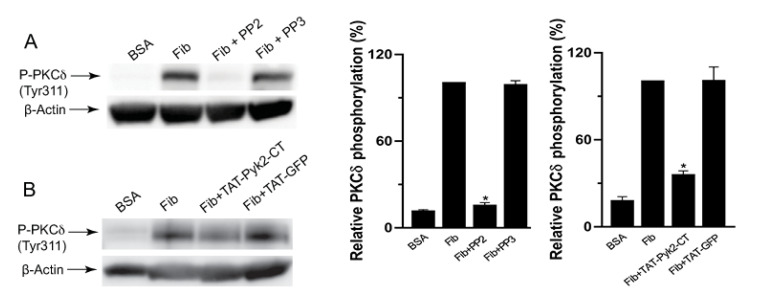
The effect of Src and Pyk2 inhibition on αIIbβ3-mediated PKCδ phosphorylation. (**A**) Fibrinogen-adherent platelets in the presence of 10 µM PP2 or 10 µM PP3 were probed with anti-phospho-PKCδ (Tyr311) or anti-β-actin antibodies. The blots shown are representative of three independent experiments and are presented as mean ± SE. * *p* < 0.05. (**B**) Fibrinogen-adherent platelets in the presence of Pyk2 inhibitor TAT-Pyk2-CT or TAT-GFP (control) were probed with anti-phospho-PKCδ (Tyr311) or anti-β-actin antibodies by Western blotting. The blots shown are representative of three independent experiments and are presented as mean ± SE. * *p* < 0.05.

Src-family kinases (SFKs) are the major tyrosine kinases downstream of integrin αIIbβ3-mediated signaling in platelets and also mediate PKCδ phosphorylation downstream of PARs in platelets [14]. To determine the molecular pathway that is responsible for integrin-mediated PKCδ phosphorylation, we first evaluated the role of Src in integrin αIIbβ3-mediated PKCδ phosphorylation. As shown in Figure 2A, PKCδ phosphorylation caused by platelet adhesion to fibrinogen was completely inhibited in the presence of Src inhibitor PP2, whereas PP3 had no effect, suggesting a role of Src in the regulation of integrin-mediated PKCδ phosphorylation. In addition, αIIbβ3-mediated phosphorylation of PKCδ was inhibited in the presence of Pyk2 selective inhibitor TAT-Pyk2-CT, whereas TAT-GFP control had no effect (Figure 2B), suggesting that integrin-mediated PKCδ phosphorylation was regulated by Pyk2.

### 2.3. The Effect of PKCδ on αIIbβ3-Mediated Pyk2 and Akt Phosphorylation

**Figure 3:**
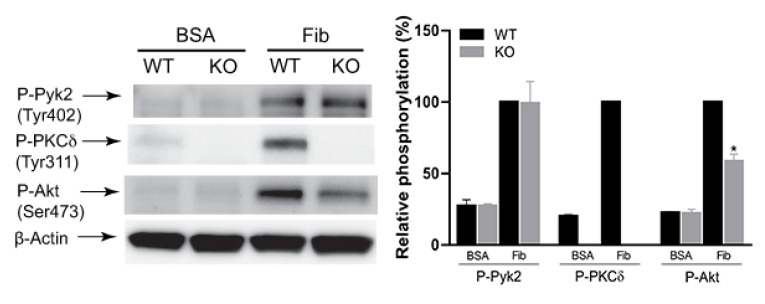
The effect of PKCδ on Pyk2 and Akt phosphorylation by αIIbβ3-mediated outside-in signaling. Washed platelets from PKCδ -/- mice and PKCδ +/+ littermates were plated on either BSA (5 mg/mL) or fibrinogen (100 μg/mL) for 45 min and probed with anti-phospho-Pyk2 (Tyr402), anti-phospho-PKCδ (Tyr311), anti-phospho-Akt (Ser473) or anti-β-actin antibodies by western blotting. The blots are representative of three experiments and are mean ± SE. * *p* < 0.05.

To determine the role of PKCδ in integrin-mediated outside-in signaling, we tested the effect of PKCδ on αIIbβ3-mediated Pyk2 and Akt phosphorylation using PKCδ-deficient platelets. As shown in Figure 3, integrin-mediated Pyk2 phosphorylation was not affected in PKCδ -/- platelets, whereas Akt phosphorylation was significantly inhibited in PKCδ -/- platelets, supporting the idea in the previous figure that PKCδ was a downstream effector of Pyk2 in outside-in signaling.

### 2.4. The Effect of PKCδ on Platelet Spreading and Clot Retraction

**Figure 4:**
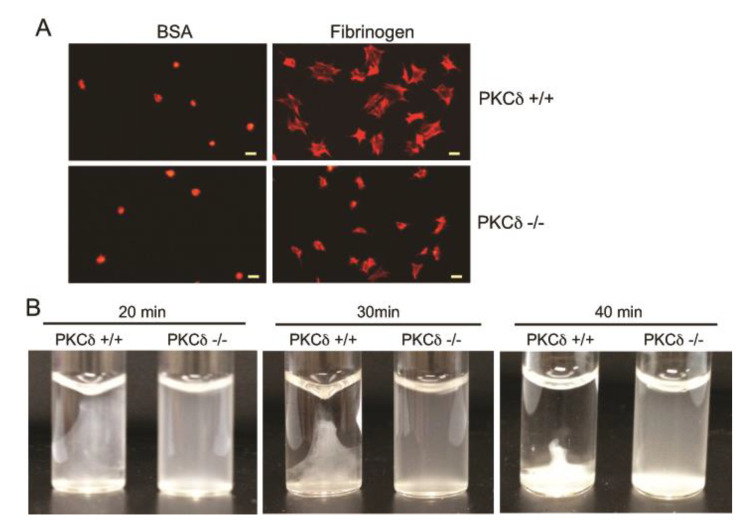
Role of PKCδ in platelet spreading on fibrinogen and clot retraction. (**A**) Washed platelets from PKCδ -/- mice and PKCδ +/+ littermates were plated on either BSA (5 mg/mL) or fibrinogen (100 μg/mL) for 45 min, stained with rhodamine phalloidin, and analyzed by fluorescence microscopy. Scale bar, 5 µm. (**B**) Platelets from PKCδ -/- mice and PKCδ +/+ littermates were mixed with CaCl_2_ (1 mM) and fibrinogen (2 mg/mL). Clot retraction was initiated by adding 0.1 U/mL of thrombin and allowed to proceed at 37 °C. Photographs of the clots were taken by a digital camera at 20, 30, and 40 min. The data shown are representative of three independent experiments.

To determine the functional role of PKCδ in outside-in signaling in platelets, we examined the effect of PKCδ on platelet spreading on immobilized fibrinogen and clot retraction using PKCδ-deficient platelets. As shown in Figure 4A, platelet spreading on immobilized fibrinogen was significantly inhibited in PKCδ-deficient platelets, indicating the contribution of PKCδ to integrin-mediated platelet spreading. Moreover, clot retraction from PKCδ -/- mice was dramatically delayed compared to PKCδ +/+ mice (Figure 4B), indicating that PKCδ plays a role in regulating αIIbβ3-dependent interactions with elements of the cytoskeleton.

## 3. Discussion

PKCδ is abundantly expressed in platelets and is activated by various agonists including thrombin, collagen, ADP, and von Willebrand factor (vWF) [16]. Regulation of PKCδ signaling was found to be complex per the identification of several potential novel phosphorylation sites [27]. Although the mechanism and the role of PKCδ activation via inside-out signaling have been extensively studied in platelets, its role and molecular mechanisms via integrin αIIbβ3-mediated outside-in signaling in platelets have not been determined. Therefore, we identified the role of PKCδ in integrin αIIbβ3-mediated outside-in signaling in platelets using pharmacological inhibitors and PKCδ-deficient mice. We demonstrated a novel outside-in signaling pathway involving sequential activation of the Src-Pyk2-PKCδ pathway downstream of integrin αIIbβ3 in platelets.

PKCδ is phosphorylated on Thr505 and Tyr565 [16,28]. It is also phosphorylated on Tyr311-mediating thromboxane generation in ADP- and PAR-stimulated platelets [13,14,29]. We first investigated whether PKCδ is phosphorylated downstream of integrin αIIbβ3-mediated outside-in signaling by stimulating the platelets with 2-MeSADP and AYPGKF. We observed that AYPGKF- and 2-MeSADP-induced PKCδ phosphorylation was significantly inhibited in the presence of fibrinogen receptor antagonist GR144053, demonstrating that PKCδ is activated downstream of integrin αIIbβ3-mediated outside-in signaling. Consistently, a previous study showed that PKCδ phosphorylation by collagen and thrombin is regulated by αIIbβ3 outside-in signaling [25]. 2-MeSADP-induced PKCδ phosphorylation was completely inhibited by GR144053, suggesting that PKCδ phosphorylation by ADP occurs in an integrin-dependent manner. GR144053 failed to completely inhibit AYPGKF-induced PKCδ phosphorylation, suggesting the involvement of both integrin-dependent and -independent pathways. Contrary to ADP, a previous study indicated that PAR agonists could induce PKCδ phosphorylation via a G_q_-mediated pathway [14]. Thus, the remaining PKCδ phosphorylation in presence of GR144053might be mediated by AYPGKF-induced G_q_-signaling. 

Integrin αIIbβ3-mediated outside-in signaling involves a number of known intracellular effectors, including SFKs, Pyk2, and Akt [5,26,30]. SFKs are known to play a central role in mediating the platelet responses and it has been demonstrated that SFK regulates tyrosine phosphorylation of PKCδ [19]. PKCδ tyrosine phosphorylation in response to GPVI agonists was also shown to be regulated by SFKs [29,31,32]. We, along with others, demonstrated that Pyk2 plays a major role in integrin outside-in signaling and might be involved in protein-tyrosine phosphorylation in platelets [26,33,34,35]. A prior study showed that Src mediates Pyk2 activation in fibrinogen-adherent platelets [34]. We demonstrated that integrin-mediated outside-in signaling from fibrinogen-adherent platelets resulted in the phosphorylation of PKCδ, confirming the activation of PKCδ by integrin αIIbβ3-mediated outside-in signaling. We observed that phosphorylation of PKCδ was completely inhibited by the Src kinase inhibitor PP2, and selective Pyk2-inhibitor TAT-Pyk2-CT also significantly inhibited PKCδ phosphorylation, suggesting that integrin αIIbβ3-mediated PKCδ is regulated by SFK and Pyk2. The residual PKCδ phosphorylation in the presence of Pyk2 inhibitor might be mediated by some other integrin-mediated Src-dependent but Pyk2-independent pathway. We observed that integrin αIIbβ3-mediated Pyk2 phosphorylation was not affected in PKCδ-deficient platelets, confirming that Pyk2 is an upstream regulator of PKCδ in integrin αIIbβ3-mediated signaling in platelets. Considering that Pyk2 was shown to be regulated by Src in integrin αIIbβ3-mediated outside-in signaling, our results indicated that integrin αIIbβ3-mediated PKCδ activation is regulated through Src- and Pyk2-dependent pathways. 

Integrin αIIbβ3-mediated outside-in signaling was found to be critically important in stable platelet adhesion, spreading, and clot retraction [36]. We observed that platelets from PKCδ-deficient mice showed a defective ability to adhere and spread on immobilized fibrinogen. Additionally, integrin αIIbβ3-mediated clot retraction was significantly inhibited in PKCδ-deficient platelets, demonstrating that PKCδ plays an important role in regulation of integrin αIIbβ3-mediated platelet adhesion, spreading and clot retraction. 

In conclusion, we demonstrated that PKCδ is activated downstream of αIIbβ3-mediated outside-in signaling in platelets. In addition, PKCδ is regulated by Src and Pyk2 in platelets. Finally, PKCδ is an important regulator of integrin αIIbβ3 outside-in signaling and contributes a major role in platelet adhesion, spreading, and clot retraction.

## 4. Materials and Methods

### 4.1. Materials

2-MeSADP, thrombin, apyrase (type V), prostaglandin E1 (PGE1), bovine serum albumin (fraction V), fibrinogen, sodium citrate, and acetylsalicylic acid were purchased from Sigma (St. Louis, MO, USA). AYPGKF was custom synthesized by Invitrogen (Carlsbad, CA, USA). Rhodamine phalloidin was from Invitrogen (Carlsbad, CA, USA). Anti-phospho-Pyk2 (Tyr402), anti-phospho-Akt (Ser473), anti-phospho-PKCδ (Tyr311), and anti-β-actin antibodies were purchased from Cell Signaling Technology (Beverly, MA, USA). PP2, PP3, and GR144053 were from Enzo Life Sciences (Plymouth Meeting, PA, USA). TAT-Pyk2-CT and TAT-GFP controls were generously provided by Xiangdong Zhu, University of Chicago (Chicago, IL, USA). Secondary antibody was from Santa Cruz Biotechnology (Santa Cruz, CA, USA). All other reagents were reagent grade. 

### 4.2. Animals

All animal experiments in this study were performed in line with the approval obtained from the Chungbuk National University Animal Ethics Committee (CBNUA-873-15-02, 1 July 2019). PKCδ-deficient mice were a generous gift from Dr. Keiko Nakayama (Tohoku University Graduate School of Medicine, Aoba-ku, Sendai, Japan). Wild-type littermates were used as controls.

### 4.3. Preparation of Murine Platelets

Murine platelets were prepared by collecting whole blood from an equal number of both male and female mice as described previously [26]. Briefly, the anti-coagulated whole blood was double-centrifuged at 100× *g* for 10 min and 400× *g* for 10 min at room temperature (RT) to obtain the platelet-rich plasma (PRP) and platelet pellet, respectively. Washed platelets were prepared by re-suspending the platelet pellet in Tyrode’s buffer and adjusted to 2 × 10^8^ cells/mL.

### 4.4. Western Blotting

Phosphorylation events were measured from AYPGKF- and 2-MeSADP-stimulated platelets or fibrinogen-adherent platelets as described previously [37]. Briefly, platelets were pre-incubated with various inhibitors in some experiments. Platelets were stimulated with agonists or were allowed to adhere to immobilized fibrinogen and 3× sodium dodecyl sulfate (SDS) buffer was added to stop the reaction. The platelet lysates were loaded onto 10% SDS/polyacrylamide gel electrophoresis and transferred to polyvinylidene difluoride (PVDF) membranes. The membranes were blocked with a blocking buffer and incubated overnight with the different primary antibodies at 4 °C. Horseradish peroxidase-labeled secondary antibodies were probed to the membranes, and immunoreactivity was detected by chemiluminescence (Fuji-Film LAS-3000 CH, Tokyo, Japan). 

### 4.5. Platelet Spreading and Clot Retraction

Washed platelets were plated on fibrinogen-coated coverslips and platelet spreading was observed as previously described [38]. Briefly, platelets on fibrinogen-coated coverslips were incubated at 37 °C for 45 min. Adhered cells were fixed, permeabilized and stained with rhodamine phalloidin. For clot retraction, platelets were added to a glass cuvette and mixed with 1 mM CaCl_2_ and 2 mg/mL fibrinogen. Clot retraction was initiated by adding 0.1 U/mL of thrombin, allowed to proceed at 37 °C, and then photographed at indicated time points.

### 4.6. Statistical Analysis

The significance of difference between data was analyzed by Student’s *t*-test using GraphPad Prism software (version 3.0) (San Diego, CA, USA). The values were shown as mean ± SE. 

## Abbreviations

PKCδ	Protein kinase C
Pyk2	Proline-rich tyrosine kinase 2
PLCγ2	Phospholipase Cγ2
TxA_2_	Thromboxane A_2_
PI3K	Phosphoinositide 3-kinase
FAK	Focal adhesion kinase
DAG	Diacylglycerol
GPVI	Glycoprotein VI
PAR	Protease-activated receptor
2-MeSADP	2-Methylthio-adenosine diphosphate
vWF	von Willebrand factor
SFKs	Src family kinases
PRP	Platelet-rich plasma
